# Malnutrition Markers and Serum Ghrelin Levels in Hemodialysis Patients

**DOI:** 10.1155/2014/765895

**Published:** 2014-10-29

**Authors:** Farzaneh Montazerifar, Mansour Karajibani, Farnia Gorgij, Ommolbanin Akbari

**Affiliations:** ^1^Pregnancy Health Research Center, Department of Nutrition, School of Medicine, Zahedan University of Medical Sciences, Zahedan 9854, Iran; ^2^Health Promotion Research Center, Department of Nutrition, School of Medicine, Zahedan University of Medical Sciences, Zahedan 9854, Iran; ^3^Department of Nutrition, School of Medicine, Zahedan University of Medical Sciences, Zahedan 9854, Iran

## Abstract

*Objective*. The aim of study was to investigate the changes levels of serum ghrelin in HD patients and its relationship to some malnutrition markers compared with healthy controls.* Methods*. Forty-five patients on hemodialysis and forty healthy controls were studied. Biochemical parameters and serum ghrelin levels were measured. Both daily dietary intakes and body mass index (BMI) assessments were performed for evaluation of nutritional status.* Results*. Ghrelin concentrations were significantly reduced in patients undergoing hemodialysis when compared to healthy controls (5 ± 0.68 (1.1–18.5) pg/mL* versus *7.8 ± 0.84 (2.4–18.3) pg/mL; *P* = 0.004). BMI and serum albumin in HD patients were markedly reduced compared to controls. The patients with an insufficient intake of energy and protein demonstrated slightly lower levels of serum ghrelin. A negative correlation between serum ghrelin concentration with age (*r* = −0.34, *P* = 0.02), BUN (*r* = −0.26, *P* < 0.01), and serum creatinine (*r* = −0.27, *P* < 0.01) was observed in HD patients.* Conclusions*. The findings suggest that decreased ghrelin levels in HD patients might be associated with anorexia. Further studies are needed to determine changes in serum ghrelin levels during dialysis and to clarify whether the decrease in ghrelin levels contributes to the malnutrition that is common in these patients.

## 1. Introduction

Protein-energy malnutrition and anorexia are common complications of uremia that occur frequently in renal failure patients [1–6]. The causes of malnutrition are not fully known. Some studies have reported that the accumulation of uremic toxic metabolites is one of the important factors [7]. The association between nutrition-regulating hormones and malnutrition is also important in HD patients [5, 6]. Ghrelin, one of the most potent orexigenic hormones, has been suggested as another possible mediator of anorexia in ESRD patients. This 28-amino acid hormone is produced largely in the stomach [1, 2, 6, 8–10]. Recent reports suggest that other tissues, including the kidney and brain, also synthesize ghrelin [9, 11]. Ghrelin is known to have multiple concurrent actions, including the anti-inflammatory effects [1, 12], release of growth hormone [2, 13, 14], fat accumulation, increased food intake, and stimulation of hypothalamic appetite centers [4, 9, 10, 13, 14]; thus it may help to regain appetite and struggle with malnutrition [1, 2, 4]. It is also involved in regulation of energy balance; thus, its dysregulation may induce obesity [6, 10, 13]. It is known that serum ghrelin level is strongly higher during fasting [8, 9, 15] and increases with weight loss [8]. It has been suggested that ghrelin to be cleared through the kidney and hemodialysis clears sufficiently ghrelin [9, 13]. Several researches compared ghrelin levels in patients treated with hemodialysis and healthy individuals that have reported conflicting results. Some studies found significantly increased ghrelin levels in HD patients [6, 9, 10, 13]. While Iglesias et al. [2] showed that patients undergoing HD had similar concentrations of ghrelin in comparison with the control group, Myrvang [16] reported that low ghrelin levels in dialysis patients increase mortality risk. In the study of Dötsch et al. [17], no effect of ghrelin on improving appetite and malnutrition was confirmed. However, the changes of serum ghrelin levels and its potential correlations with markers of nutrition in hemodialysis patients are still incompletely characterized. This study investigated the changes levels of serum ghrelin in ESRD patients receiving dialysis treatment and its relationship to some malnutrition markers compared with healthy controls.

## 2. Material and Methods

In a cross sectional study, 45 ESRD patients undergoing hemodialysis (*n* = 20 males, *n* = 25 females, aged 43.2 ± 13.1 years), referred to Hemodialysis Center of Imam Ali and Khatam-Al-Anbia Hospitals, Zahedan, Iran, and 40 healthy subjects (*n* = 17 male, *n* = 23 female, aged 38 ± 12.6 years), matched for sex and age, were selected between February 2014 and May 2014. The patients underwent dialysis three times a week (each session 3-4 hours) for at least three months with a minimum *Kt*/*V* of 1.2. All participants were older than 18 years; none of them took the lipid-lowering medications and corticosteroids. During the previous three months they were not hospitalized or had not infectious and inflammatory diseases, diabetes mellitus, cardiovascular (CVD) and liver disease, thyroid, or cancer. The causes of ESRD included blood pressure (*n* = 16), kidney stone (*n* = 4), lupus erythematosus (*n* = 2), polycystic kidney (*n* = 4), glomerulonephritis (*n* = 8), and unknown (*n* = 11).

Dry body weight after dialysis and height were measured. Body mass index (BMI), as one of the markers of nutritional status, was evaluated based on the calculation of dry weight (Kg)/height² (m²) [18, 19]. BMI was categorized in accordance with the recommendations of the World Health Organization [18]. Mean daily caloric and protein intake was estimated using 24-hour recall form by a specialized dietitian and analyzed by food composition analysis software.

After an overnight fasting, blood samples were collected from all healthy controls and in the patients immediately before HD. Biochemical parameters including blood glucose, blood urea nitrogen (BUN), uric acid, creatinine, cholesterol, triglyceride, LDL, HDL, and serum albumin concentrations were determined with standard techniques by automatic analyzer. Serum albumin < 3.5 g/dL was also considered as a predictor of protein malnutrition [18].

Serum Ghrelin levels were assessed using commercial ELISA kit (Human Acylated Ghrelin ELISA kit, (BioVendor (Cat no: RA 194062400R), USA)). The serum samples were immediately frozen at −70°C until analysis.

This study was approved by the Ethics Committee of the Zahedan University of Medical Sciences (approval date: 4 January 2014; number: 6697). All subjects gave informed consent for the participation in the study.

## 3. Statistical Analysis

SPSS statistical software package program (version 18 for windows, Chicago, USA) was used for analyses. Data were expressed as mean ± SD and mean ± SEM with range and frequency, as appropriate. Variables with normal distribution were compared by Student's *t*-test, one-way ANOVA. The differences among two groups after being categorized based on the BMI, serum albumin, and age were analyzed by 2-way ANOVA for repeated measures analysis of variance using Bonferroni test. Mann-Whitney *U* and Kruskall-Wallis tests were performed for nonnormal distribution variables. The variables associated with serum ghrelin levels were first evaluated by spearman correlation coefficient. The variables that significantly correlated with serum ghrelin were assessed as independent variables in the multiple regression analysis. A *P* value < 0.05 was considered significant.

## 4. Results

Demographic and nutritional parameters of subjects are demonstrated in Table 1. The patients underlying hemodialysis had markedly lower weight (*P* < 0.0001) and BMI (*P* < 0.001) than control subjects.

Table 2 describes chemical parameters and ghrelin levels in both groups. Serum albumin levels and hemoglobin were significantly reduced (*P* < 0.0001), and BUN, serum creatinine (*P* < 0.0001), and uric acid (*P* = 0.007) levels were markedly increased when compared to healthy controls (*P* < 0.0001). No significant difference was found between patients and controls for serum lipid profile levels. Ghrelin concentration was significantly declined in HD patients when compared to healthy controls (5 (1.1–18.5) pg/mL* versus* 7.8 (2.4–18.3) pg/mL; *P* = 0.004).

Table 3 demonstrates ghrelin levels based on malnutrition markers in both groups. Bonferroni's multiple comparison test revealed that the levels of serum ghrelin were significantly lower in overweight/obese patients with BMI ≥ 25 kg/m^2^ (5.2 (1.1–16.1)* versus* 8 (3.3–18.3); *P* < 0.01) and nonobese patients with BMI < 25 kg/m^2^ (4.9 (1.1–18.5)* versus* 7.8 (2.4–16.9); *P* < 0.05) compared to control subjects. The levels of this peptide in patients with BMI less than 25 kg/m^2^ were modestly lower when compared to obese patients (*P* > 0.05).

Older patients (>50 years old) had moderately (not significant) lower serum ghrelin levels compared to those with less than 50 years and control subjects (3.8 (1.1–15.6)* versus* 5.8 (1.1–18.5); *P* = 0.055 and 7 (2.4–18.3); *P* = 0.06, resp.).

Serum ghrelin levels were markedly lower in patients with serum albumin < 3.5 g/dL than patients with serum albumin > 3.5 g/dL and controls (3.8 (1.2–9.7)* versus* 6.1 (1.1–18.5) (*P* < 0.04) and* versus* 7.8 (2.4–18.3), (*P* < 0.001), resp.), but no significant differences were observed between patients with serum albumin > 3.5 g/dL and control subjects.

When obese and nonobese patients were analyzed separately, energy (kcal/day), protein, and fat (g/day) intakes were markedly lower in both patients groups than controls (1321.5 ± 618* versus* 1839 ± 587 kcal/day; *P* < 0.001 and 1267 ± 550* versus *1659 ± 665 kcal/day; *P* < 0.001) (54 ± 29* versus *84 ± 32 g/day; *P* < 0.001 and 59 ± 21.6* versus *83.6 ± 25 g/day; *P* < 0.001) and (45.5 ± 19* versus *61 ± 32 g/day; *P* < 0.05 and 41 ± 20* versus *54.5 ± 29 g/day; *P* < 0.05, resp.), as described in Table 4.

A negative correlation between serum ghrelin concentration with age (*r* = −0.34, *P* = 0.02), BUN (*r* = −0.26, *P* < 0.01), and serum creatinine (*r* = −0.27, *P* < 0.01) was observed among HD patients. Multivariate regression analysis demonstrated a significant negative correlation between serum ghrelin levels with age (*β* = −0.269, *P* = 0.02). In the multivariate model, serum albumin level as dependent variable correlated with BUN (*β* = −0.492, *P* < 0.01) and creatinine (*β* = −0.557, *P* < 0.01) (Table 5).

## 5. Discussion

We studied serum ghrelin levels in renal failure patients undergoing dialysis in comparison to healthy controls. Our data demonstrated a significantly decreased serum ghrelin level in ESRD patients treated by hemodialysis, which might be attributed to the kidney dysfunction in synthesizing it. On the other hand, the dialysis also degrades and/or is clear of ghrelin [9, 13]. This result is in accordance with Myrvang [16] study but incompatible with those of other studies which have previously been described in renal failure patients [2, 3, 6, 9, 10]. To assess whether ghrelin levels are associated with malnutrition in HD patients, some nutritional and laboratory markers affecting malnutrition including food intakes, BMI, serum albumin, BUN, creatinine, uric acid, and lipid profile were assessed in these patients. In our study, BMI in patients underlying hemodialysis was markedly reduced compared to controls. This finding was similar to Şahin et al. [20] results but was inconsistent with Pérez-Fontán et al. [6] results. In agreement to earlier studies [7, 9] serum albumin level was notably lower than controls. Low serum albumin, one of the important nutritional predictors, is associated with mortality and morbidity in renal failure patients [7, 20, 21]. Moreover, some authors have demonstrated that a reduction in body weight increases ghrelin concentrations, suggesting that these patients have some forms of ghrelin resistance; thus, they have a tendency to develop anorexia [19, 22]. In our study, patients with BMI < 25 kg/m² and serum albumin <3.5 g/dL detected moderately (not significant) decreased levels of serum ghrelin more than obese patients with serum albumin >3.5 g/dL and control subjects. Inconsistent with earlier studies, no correlation between serum ghrelin with BMI [6, 10, 23–25] and serum albumin [7] was found in this study.

Although ghrelin is postulated to stimulate food intake, statistically no correlation was found between serum ghrelin levels and dietary and energy intakes in the present study. This finding was inconsistent with previous studies [14, 19]. Food intakes were markedly lower in both obese and nonobese HD patients as compared to controls. The patients with a low intake of energy had slightly (not significant) lower levels of serum ghrelin (data not shown). A study showed that 70% of the HD patients consumed food less than recommended levels [19]. Another research revealed that energy intake was notably lower in anorexia HD patients than in nonanorexia patients [26]. Similar to our study, Refael et al. [27] showed that uremic patients with anorexia had lower ghrelin levels compared to obese or normal appetite subjects. They suggested that, in adult uremic patients because of insulin and growth hormone metabolism disorders, ghrelin may modify energy intake. Some studies have also represented that ghrelin alone could not be associated with renal function, and acylated (active) ghrelin “as a vital part of ghrelin,” which is known to increase food intake in humans and rats [9, 10, 19, 26] and exerts orexigenic effects [14], should be investigated in uremic patients. However, in contrast to previous studies [10, 16, 28, 29], our findings depict a negative energy balance associated with malnutrition, which may be caused by reducing the concentration of ghrelin in HD patients, suggesting that probably the ghrelin system is highly unsuccessful in regulating the nutritional abnormalities in these patients.

After multivariate regression analysis, age was the variable correlated with serum ghrelin in HD patients. Similar to previous studies, in which age was considered as inverse predictor of ghrelin both in patients and in control subjects [6, 30], our study also showed that older patients had slightly lower serum ghrelin levels compared to those with less than 50 years and control subjects. The findings suggest that ghrelin level alterations might be associated with undernutrition in aging. However, some studies did not show relevant correlation between two variables [3, 10].

In addition, uremic toxins including BUN and creatinine are also important factors which are associated with anorexia in these patients [7, 10]. A negative correlation observed between serum ghrelin and albumin levels with BUN and creatinine in this study, suggesting that high level of uremic toxin metabolites may lead to decreased serum ghrelin level or impair ghrelin's function, causes anorexia and malnutrition in these patients.

The limitations of our study were small sample size, lack of longitudinal data, and failure to measure the levels of protein catabolic rate (PCR), metabolic acidosis, bicarbonate, and chloride, which may also affect appetite and possibly the level of ghrelin in HD patients.

## 6. Conclusion

The findings suggest that decreased ghrelin levels in HD patients might be associated with high anorexia. However, this study could not explain the likely role of ghrelin in low food intake. Further studies are needed to determine changes in serum ghrelin levels during dialysis and to clarify whether the decrease in ghrelin levels contributes to the malnutrition that is common in these patients.

## Figures and Tables

**Table 1:** Characteristics of subjects.

Groups variables	HD patients (*n* = 45)	Controls (*n* = 40)	Pv
Age (yr)	43.2 ± 13.1	38 ± 12.6	NS
Sex (M/F)	20/25	17/22	
Dry weight (Kg)	55.5 ± 14.4	71.6 ± 17	0.0001
BMI (Kg/m²)	22 ± 2.1	25.7 ± 4.9	0.001
Duration of dialysis (months)	38.5 ± 25.2 (3–96)	—	
*Kt*/*V*	1.7 ± 0.8	—	
Blood pressure (mmHg)			
Systolic	124.2 ± 11	122 ± 10.5	NS
Diastolic	79.3 ± 9 .2	80.1 ± 7.8	NS

NS: not significant.

**Table 2:** Chemical parameters of HD patients and controls.

Groups variables	HD patients (*n* = 45)	Controls (*n* = 40)	Pv
BUN (mg/dL)	60.4 ± 24.5	12.8 ± 3.3	0.0001
Uric acid (mg/dL)	6.3 ± 1.7	5.7 ± 1.5	0.007
Creatinine (mg/dL)	8.7 ± 3.6	0.78 ± 0.18	0.0001
Cholesterol (mg/dL)	168 ± 79	169 ± 42.6	NS
LDL-C (mg/dL)	82.2 ± 24	82.3 ± 23.7	NS
HDL-C (mg/dL)	42.4 ± 14.3	42.7 ± 12.4	NS
Triglyceride (mg/dL)	129 ± 81.6	119 ± 79.4	NS
Albumin (g/dL)	3.4 ± 0.4	4.5 ± 0.76	0.0001
Sodium (mmol/L)	141 ± 12	139 ± 10	NS
Potassium (mmol/L)	5 ± 0.6	4.7 ± 0.9	NS
Calcium (mg/dL)	8.7 ± 1.1	9 ± 1.4	NS
Phosphorous (mg/dL)	5.2 ± 1.4	4.9 ± 1.1	NS
Hemoglobin	10 ± 2.3	13.7 ± 1.9	0.0001
^*^Ghrelin (pg/mL)	5 ± 0.68 (1.1–18.5)	7.8 ± 0.84 (2.4–18.3)	0.004

Data were expressed as mean ± SD.

BUN: blood urea nitrogen; LDL-C: low density lipoprotein-cholesterol; HDL-C: high density lipoprotein.

^*^Ghrelin level was reported as mean ± SEM and range because the data were not normally distributed.

NS: not significant.

**Table 3:** Serum ghrelin levels based on body mass index (BMI), age, and serum albumin levels in HD patients and controls.

Groups variables	HD patients (*n* = 45)	Control (*n* = 40)	Pv
BMI (Kg/m²)			
<25	4.9 (1.1–18.5) (*n* = 34)	7.8 (2.4–16.9) (*n* = 20)	0.01
≥25	5.2 (1.1–16.1) (*n* = 11)	8 (3.3–18.3) (*n* = 20)	0.04
Pv	0.17	0.5	
Age (yrs)			
<50	5.8 (1.1–18.5) (*n* = 27)	7 (2.4–18.3) (*n* = 25)	0.06
>50	3.8 (1.1–15.6) (*n* = 18)	6.5 (3.5–10.2) (*n* = 15)	
Pv	0.055	0.17	
Albumin			
<3.5	3.8 (1.2–9.7) (*n* = 5)	— (*n* = 0)	
>3.5	6.1 (1.1–18.5) (*n* = 40)	7.8 (2.4–18.3) (*n* = 40)	0.001
Pv	0.04	—	

**Table 4:** Food intakes in HD patients and controls.

BMI (kg/m²)	HD patients		Controls	
	<25 (*n* = 34)	≥25 (*n* = 11)	<25 (*n* = 20)	≥25 (*n* = 20)
Energy (Kcal/day)	^*^1267 ± 550	^*^1321.5 ± 618	1659 ± 665	1839 ± 587
Carbohydrate (g/day)	193 ± 84	200 ± 97	214 ± 94	237 ± 106
Protein (g/day)	^*^59 ± 21.6	54 ± 29	83.6 ± 25	84 ± 32
Fat (g/day)	^¶^41 ± 20	^¶^45.5 ± 19	54.5 ± 29	61 ± 32

Data were expressed as mean ± SD.

^*^ *P* < 0.001 HD patients versus controls.

^¶^ *P* < 0.05 HD patients versus controls.

**Table 5:** Multivariate regression analysis between serum ghrelin and albumin levels with various parameters in HD patients.

Parameters		Regression coefficient (beta)	Pv
Ghrelin	Age	−0.269	0.02

Albumin	BUN	−0.492	0.01
	Creatinine	−0.557	0.01
